# Novel Morphological Features on CMR for the Prediction of Pathogenic Sarcomere Gene Variants in Subjects Without Hypertrophic Cardiomyopathy

**DOI:** 10.3389/fcvm.2021.727405

**Published:** 2021-09-17

**Authors:** Nikki van der Velde, Roy Huurman, H. Carlijne Hassing, Ricardo P. J. Budde, Marjon A. van Slegtenhorst, Judith M. A. Verhagen, Arend F. L. Schinkel, Michelle Michels, Alexander Hirsch

**Affiliations:** ^1^Department of Cardiology, Erasmus Medical Center, University Medical Center Rotterdam, Rotterdam, Netherlands; ^2^Department of Radiology and Nuclear Medicine, Erasmus Medical Center, University Medical Center Rotterdam, Rotterdam, Netherlands; ^3^Department of Clinical Genetics, Erasmus Medical Center, University Medical Center Rotterdam, Rotterdam, Netherlands

**Keywords:** hypertrophic cardiomyopathy, cardiovascular magnetic resonance, sarcomere gene variants, morphological features, genotype risk prediction

## Abstract

**Background:** Carriers of pathogenic DNA variants (G+) causing hypertrophic cardiomyopathy (HCM) can be identified by genetic testing. Several abnormalities have been brought forth as pre-clinical expressions of HCM, some of which can be identified by cardiovascular magnetic resonance (CMR). In this study, we assessed morphological differences between G+/left ventricular hypertrophy-negative (LVH-) subjects and healthy controls and examined whether CMR-derived variables are useful for the prediction of sarcomere gene variants.

**Methods:** We studied 57 G+ subjects with a maximal wall thickness (MWT) < 13 mm, and compared them to 40 healthy controls matched for age and sex on a group level. Subjects underwent CMR including morphological, volumetric and function assessment. Logistic regression analysis was performed for the determination of predictive CMR characteristics, by which a scoring system for G+ status was constructed.

**Results:** G+/LVH- subjects were subject to alterations in the myocardial architecture, resulting in a thinner posterior wall thickness (PWT), higher interventricular septal wall/PWT ratio and MWT/PWT ratio. Prominent hook-shaped configurations of the anterobasal segment were only observed in this group. A model consisting of the anterobasal hook, multiple myocardial crypts, right ventricular/left ventricular ratio, MWT/PWT ratio, and MWT/left ventricular mass ratio predicted G+ status with an area under the curve of 0.92 [0.87–0.97]. A score of ≥3 was present only in G+ subjects, identifying 56% of the G+/LVH- population.

**Conclusion:** A score system incorporating CMR-derived variables correctly identified 56% of G+ subjects. Our results provide further insights into the wide phenotypic spectrum of G+/LVH- subjects and demonstrate the utility of several novel morphological features. If genetic testing for some reason cannot be performed, CMR and our purposed score system can be used to detect possible G+ carriers and to aid planning of the control intervals.

## Introduction

Hypertrophic cardiomyopathy (HCM) is the most common genetic heart disease, which is caused by sarcomere gene variants in up to 60% of the cases (1). HCM is most commonly inherited in an autosomal dominant fashion. Therefore, relatives of HCM patients are offered genetic testing, which enables identification of carriers of pathogenic DNA variants, even before manifestation of left ventricular hypertrophy (LVH). These genotype-positive (G+)/LVH- subjects are then subject to routine cardiac evaluation to monitor phenotypic progression (1–4). Several abnormalities have been brought forth as pre-clinical expressions of HCM, ranging from electrocardiographic changes and diastolic dysfunction to myocardial crypts and anterior mitral valve leaflet (AMVL) elongation (5–9). In recent years, Cardiovascular Magnetic Resonance (CMR) has emerged as a valuable technique in the diagnosis and follow-up of HCM, owing to the superior assessment of cardiac morphology and function compared to transthoracic echocardiography as well as the possibility of tissue characterization (10). When genetic testing is refused by relatives of HCM patients or is impractical/impossible, genotype risk prediction from pre-clinical expressions of HCM may be useful. In this present study, we assessed morphological and functional differences between a cohort of genotype-positive (G+)/LVH- subjects and healthy controls and examined whether CMR-derived variables are useful for the prediction of pathogenic DNA variants.

## Materials and Methods

### Study Population

For this single-center case-control study, G+/LVH- subjects who underwent CMR were selected from the Inherited Cardiomyopathy registry of the Erasmus Medical Center, Rotterdam, The Netherlands. G+ subjects emerged from genetic cascade screening in first-degree relatives of HCM patients at the cardiogenetic outpatient clinic and were included in case of likely pathogenic (class 4) or pathogenic (class 5) DNA variants, in accordance with the American College of Medical Genetics and Genomics recommendations (11). The cardiogenetic testing procedure has been previously described (12). CMR imaging was performed between October 2008 and September 2020. LVH- was defined as a maximal wall thickness (MWT) <13 mm on CMR.

The control group consisted of non-related healthy controls matched for age and sex on a group level, which were free of cardiovascular disease. Genetic status with respect to pathogenic DNA variants causing HCM was not determined in these healthy controls. They underwent CMR imaging between June 2018 and November 2019.

This study conforms to the principles of the Declaration of Helsinki. All G+/LVH- subjects gave informed consent for inclusion in the registry, and the study in these subjects did not meet the requirements of a study subject according to the Medical Research Involving Human Subjects Act. The study in healthy controls was approved by the local Institutional Review Board (MEC-2014-096). Written informed consent was obtained from all G+/LVH- subjects and healthy controls.

### Cardiovascular Magnetic Resonance

CMR in G+/LVH- subjects was performed on a clinical 1.5T (*n* = 51) or 3T (*n* = 6) MRI systems (SIGNA Artist *n* = 44, Discovery MR750: *n* = 5; SIGNA HDxt: *n* = 4; Discovery MR450: *n* = 3; SIGNA Premier: *n* = 1; GE Healthcare, Milwaukee, WI, USA). CMR imaging in all 40 healthy controls were performed on the same clinical 1.5T SIGNA Artist system. Scans were performed using a dedicated cardiac/anterior array coil, electrocardiographic gating and breath-hold techniques. The protocol included steady-state free precession (SSFP) cine imaging. Pre- and/or post-contrast T1-mapping was performed in a subset of G+/LVH- subjects and healthy controls that were scanned on the SIGNA Artist.

**Figure 1:**
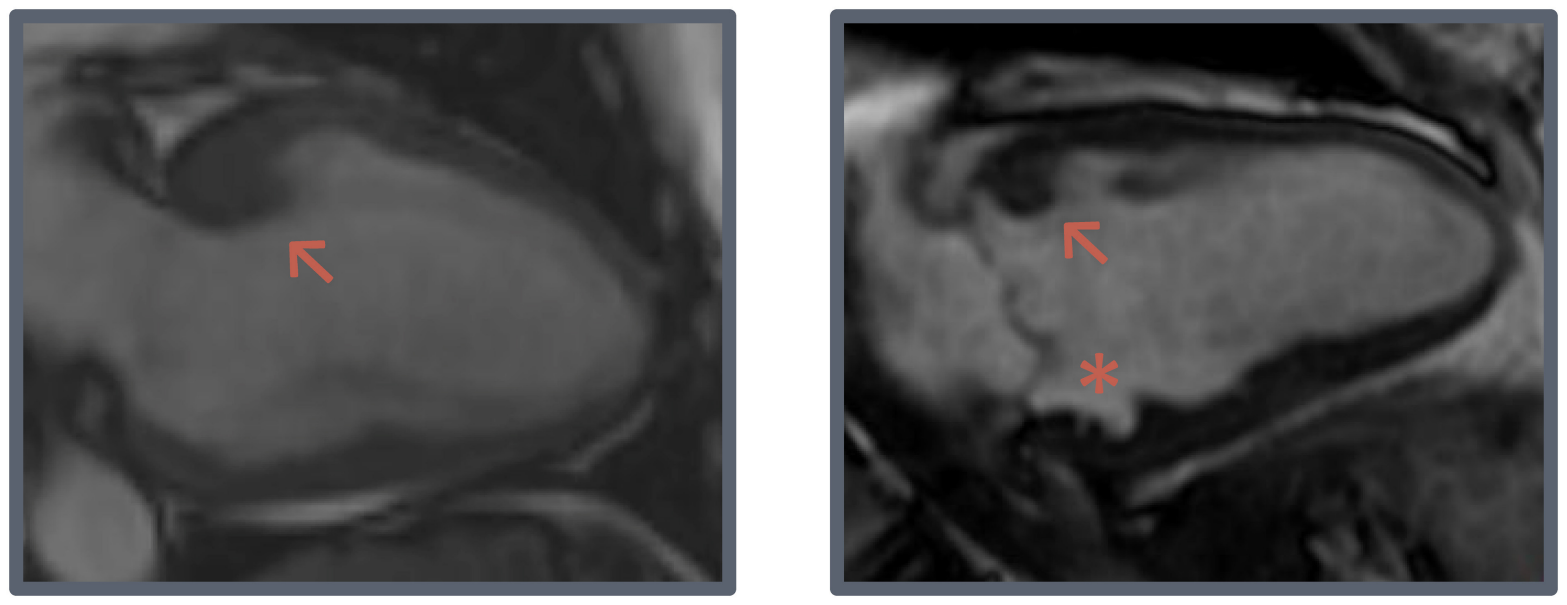
Hook-shaped configurations. Two examples of prominent hook-shaped configurations of the anterobasal segment (arrow) and a myocardial crypt (asterisk).

SSFP cine images were obtained during breath-hold in a contiguous stack of short-axis (SA) views, with coverage from base to apex, and in all three long-axis views (2-, 3-, and 4-chamber). Typical SSFP cine imaging scan parameters were: slice thickness 6–8 mm, interslice gap 2–4 mm, TR/TE 3.5–4.5/1.4–2.0 ms, flip angle 45–85°, ASSET 2, field of view 280–380 × 250–340 mm and acquired matrix 192–280 × 160–256. CMR analysis was performed on anonymized images by an experienced CMR reader with 4 years of experience (NV). Functional analysis was performed on SA images by manually drawing epi- and endocardial contours in end-systolic and end-diastolic phase, with inclusion of papillary muscles and trabeculations in the left ventricular (LV) volume. MWT, thickness of the interventricular septum (IVS) and posterior wall (PWT) were measured on a basal SA cine image in end-diastolic phase. LV mass (LVM) was determined and was normalized for sex by dividing body surface area-adjusted values by the mean normal value for men and women separately (LVM_i,n_) (13). Morphological features such as myocardial crypts, hypertrabeculation, anterobasal hook, and AMVL elongation were also assessed on SSFP images. Myocardial crypts were defined as myocardial recess of ≥50% the depth of the adjacent myocardial tissue. Hypertrabeculation was measured by determining the ratio between non-compacted and compacted myocardium according to the Petersen criteria: end-diastolic non-compacted/compacted ratio >2.3 in any long-axis view (14). The anterobasal hook was defined as a prominent, isolated, focal hook-shaped configuration of the anterobasal segment with relatively thin adjacent myocardium and a ratio of the MWT of the basal segment and the adjacent myocardium of ≥2. This was assessed on the long-axis 2-chamber view in end-diastole (Figure 1) (15). AMVL length was measured in 3-chamber view, in the phase where the AMVL was most visible, in the mid- to end-diastolic phase, from the most distal part of the leaflet to its insertion in the posterior aortic wall.

T1 mapping was performed in a basal SA view using a modified look-locker inverse recovery sequence with a 5(3)3 acquisition scheme pre-contrast and a 4(1)3(1)2 acquisition scheme post-contrast (0.2 mmol/kg, Gadovist, Bayer, Mijdrecht, The Netherlands). Typical T1 mapping scan parameters were: slice thickness 8 mm, TE/TR 1.5–1.7/3.5–3.7, flip angle 35°, ASSET 2, field of view 320–400 × 270–400 mm, and acquired matrix 192 × 140. Native T1 values and extracellular volume (ECV) were measured by manually drawing a septal region of interest whereby partial volume was taken into account. Motion correction was performed. Hematocrit was determined for the calculation of extracellular volume (16). In the G+/LVH- subjects, late gadolinium enhancement (LGE) imaging was performed at least 10–15 min after intravenous administration of a gadolinium-based contrast agent, using a breath-held two-dimensional segmented inversion-recovery gradient-echo pulse sequence. Images were obtained in all long-axis views and SA views. If necessary, the preset inversion time was adjusted to null normal myocardium for LGE imaging. LGE was visually scored as presence or absence, and if applicable the pattern and localization was assessed. No LGE imaging was performed in healthy controls.

Dedicated software was used for all these measurements (Qmass software version 8.1 and Qmap T1 software version 2.2.38, Medis, Leiden, the Netherlands).

### Statistical Analysis

Values were expressed as mean ± standard deviation, median [25th−75th percentile] or number (%). Continuous data were assessed for normality, and were analyzed using the Student's *t* test or Mann-Whitney U test, as appropriate. Predictors of G+ status were identified using Firth's bias-reduced logistic regression to account for data separation. All variables were potential candidates for the models, and variable selection for the final model was done by comparing Akaike Information Criterion values, with the highest number of potential independent variables set at 5, to lessen the chance of over-fitting. All model assumptions were met. Particularly, there was no multi-collinearity. Receiver operator characteristic (ROC) curves were constructed for each variable to determine optimal cut-off values for prediction of G+ status using Youden's index, and for the full model to determine its potential use in predicting pathogenic DNA variants. A scoring system was constructed based on all relevant dichotomized predictors with weights based on regression coefficients. *P* < 0.05 were considered statistically significant. All statistical analyses were performed using SPSS version 25 (IBM Corp., Armonk, New York) and R version 3.6.1 (https://cran.r-project.org/).

## Results

An overview of baseline and genotype characteristics of the 57 G+/LVH- subjects and 40 healthy controls are shown in Table 1. This table also summarizes CMR characteristics of both groups. Overall, the median age was 45 [32–53] years and 34% were male. The groups were similar with respect to demographic characteristics. Most G+/LVH- subjects were carriers of likely pathogenic or pathogenic MYBPC3 (*n* = 42, 74%), MYH7 (*n* = 7, 12%), and MYL2 (*n* = 6, 11%) sarcomere gene variants. The genotype per individual G+/LVH- subject is shown in Supplementary Table 1.

**Table 1:** Baseline, genetics, and imaging characteristics in genotype-positive/left ventricular hypertrophy negative subjects compared to healthy controls.

	**G+/LVH- subjects**	**Healthy controls**	***p***
	**(*n* = 57)**	**(*n* = 40)**	
**Demographics**			
Age (years)	45 [31–52]	45 [32–55]	0.61
Male sex	18 (32%)	15 (38%)	0.55
Body mass index (kg/m^2^)	24 [22–27]	23 [22–25]	0.17
Ethnicity			0.30
Caucasian	56 (98%)	37 (93%)	
Other	1 (2%)	3 (8%)	
**Genetics**			
Genotype			
MyBPC3	42 (74%)		
MYH7	7 (12%)		
MYL2	6 (11%)		
TNNT2	1 (2%)		
ACTN2	1 (2%)		
**CMR characteristics**			
Left atrial volume (ml/m^2^)	44 ± 11	46 ± 11.5	0.60
LV end-diastolic volume (ml/m^2^)	80 ± 12	82 ± 12	0.44
LV end-systolic volume (ml/m^2^)	30 ± 6	33 ± 7	0.09
LV ejection fraction (%)	62 ± 5	60 ± 5	0.06
LV mass (g/m^2^)	46 ± 8	54 ± 9	<0.001
RV end-diastolic volume (ml/m^2^)	84 ± 15	93 ± 15	0.01
EV end-systolic volume (ml/m^2^)	35 ± 10	43 ± 9	<0.001
RV Ejection fraction (%)	58 ± 6	53 ± 4	<0.001
RV/LV ratio	1.05 ± 0.10	1.1 ± 0.09	<0.001
Native septal T1 (ms)^*^	975 ± 38	961 ± 27	0.06
ECV basal septum (%)^†^	29 ± 4	29 ± 4	0.85
Presence of late gadolinium enhancement^‡^	5 (9%)	N/A	N/A
Midmyocardial at hinge points	5 (9%)	N/A	N/A
Crypts	27 (47%)	5 (13%)	<0.001
≥2 crypts	9 (16%)	0 (0%)	0.01
Ratio of non-compacted/compacted myocardium >2.3	9 (16%)	4 (10%)	0.41
Hook-shaped thickening basal anterior wall	14 (25%)	0 (0%)	0.001
AMVL length (mm/m^2^)	12 ± 2	12 ± 2	0.57
Interventricular septum (mm)	8.7 ± 1.4	8.7 ± 1.6	0.96
Maximal wall thickness (mm)	10.7 ± 1.4	10.2 ± 1.4	0.07
Posterior wall thickness (mm)	6.7 ± 1.3	7.7 ± 1.3	<0.001
Interventricular septum/posterior wall ratio	1.3 ± 0.3	1.2 ± 0.2	0.001
Maximal wall thickness/posterior wall ratio	1.7 ± 0.4	1.3 ± 0.2	<0.001
Maximal wall thickness/normalized LVM_i,n_	11.4 ± 2.2	9.4 ± 1.4	<0.001

*Comparison of baseline, genetics and CMR characteristics of G+/LVH− population and healthy controls*.

*AMVL, Anterior mitral valve leaflet; CMR, cardiovascular magnetic resonance; ECV, Extracellular volume; G+, Genotype-positive; LV, Left ventricle; LVH-, Left ventricular hypertrophy negative; LVM_i,n_, Left ventricular mass indexed by body surface area and normalized by sex; RV, Right ventricle*.

* *Data available in 44/57 G+/LVH- subjects and in 40/40 healthy controls, respectively*.

† *Data available in 21/57 G+/LVH- subjects and in 26/40 healthy controls, respectively*.

‡ *Data available in 54/57 G+/LVH- subjects*.

With regard to morphological and functional parameters, both groups were similar with respect to left atrial and LV indexed dimensions and function. Remarkably, LVM_i,n_ was significantly lower in G+/LVH- subjects compared to healthy controls (0.96 ± 0.14 vs. 1.09 ± 0.14, *p* < 0.001). Similarly, G+/LVH- subjects had significant smaller indexed right ventricular (RV) volumes, which resulted in a lower RV/LV ratio (1.05 ± 0.10 vs. 1.1 ± 0.09, *p* < 0.001).

PWT was lower in G+/LVH- subjects (6.7 ± 1.3 mm vs. 7.7 ± 1.3 mm, *p* < 0.001), resulting in a higher IVS/PWT ratio and MWT/PWT ratio (1.3 ± 0.3 vs. 1.2 ± 0.2, *p* = 0.001; 1.7 ± 0.4 vs. 1.3 ± 0.2, *p* < 0.001). In G+/LVH- subjects, we observed discrepancies between the MWT and thickness of the other segments, particularly the posterior wall, seemingly due to relative myocardial thinning rather than absolute hypertrophy. We quantified this discrepancy by dividing MWT by LVM_i,n_, which was significantly higher in G+/LVH- subjects (11.4 ± 2.2 mm vs. 9.4 ± 1.4 mm, *p* < 0.001). Furthermore, we observed a prominent focal hook-shaped configuration of the anterobasal segment, which was relatively thick compared to the adjacent myocardium (Figure 1). This anterobasal hook was present in 25% of G+/LVH- subjects and in none of the healthy controls. Myocardial crypts were more often demonstrated in G+/LVH- subjects in comparison with healthy controls (47 vs. 13%, *p* < 0.001); however, ≥2 crypts was only observed in the G+/LVH- subjects (16 vs. 0%, *p* < 0.01). Native T1 and ECV were measured in a subset of G+/LVH- subjects and healthy controls (all scanned on SIGNA Artist 1.5T, native T1: *n* = 44 vs. *n* = 40; ECV: *n* = 21 vs. *n* = 26, respectively). No differences in these measurements were found between groups. A total of 5 of the 54 G+/LVH- subjects showed LGE on their CMR examination; all of them showed mid-myocardial LGE at the hinge points.

### Genotype Risk Prediction Model

**Figure 2:**
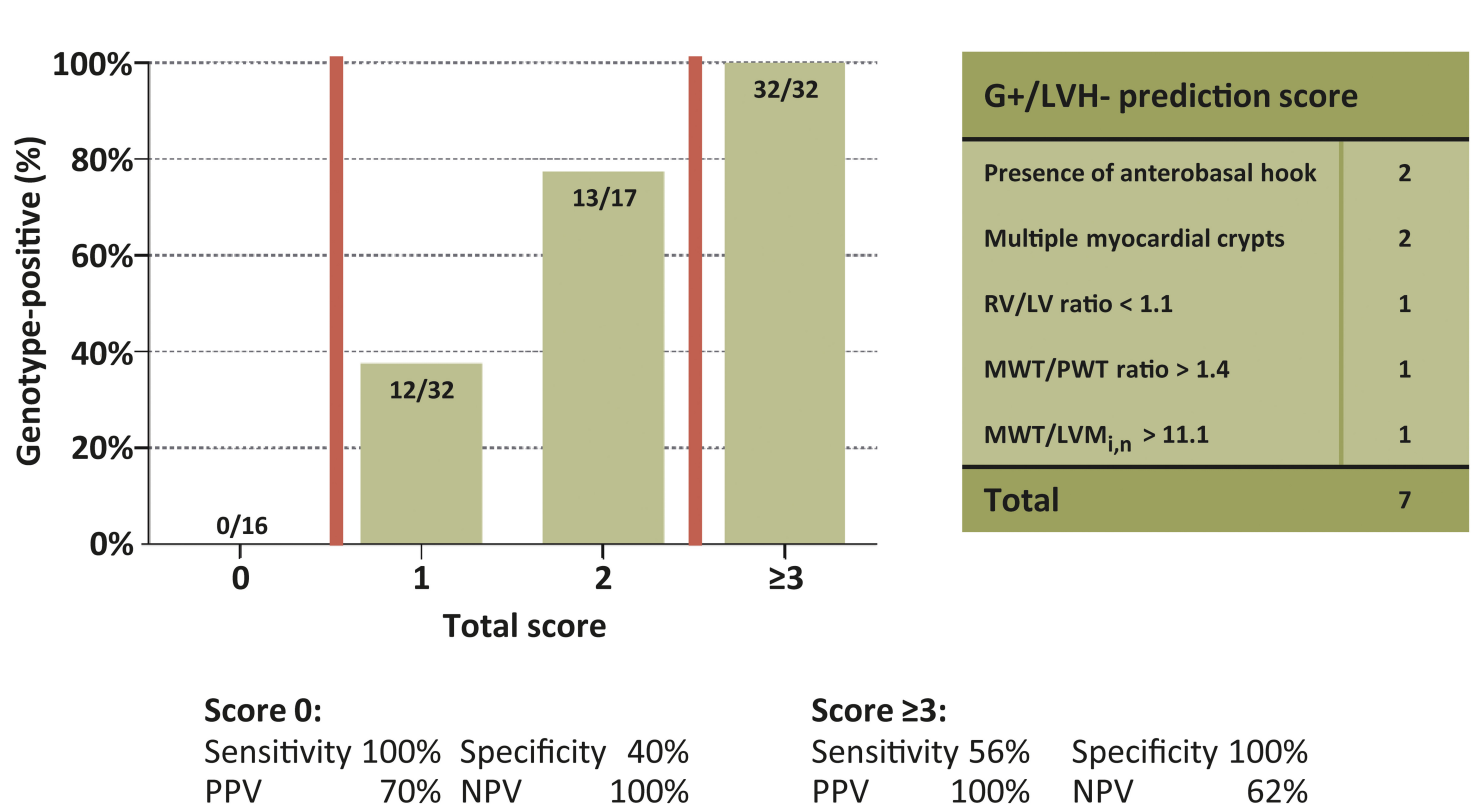
Yield of genotype risk prediction score in genotype-positive/left ventricular hypertrophy negative subjects. Proportion of G+ subjects for every score subgroup. Groups 3–7 collapsed. G+, Genotype-positive; LV, Left ventricle; LVH, left ventricular hypertrophy; LVM_i,n_, Left ventricular mass indexed by body surface area and normalized by sex; MWT, Maximal wall thickness; NPV, Negative predictive value; PPV, Positive predictive value; PWT, posterior wall thickness; RV, Right ventricle.

Univariable and multivariable linear regression analyses were performed to assess which CMR parameters were predictive of genotype-positive status (Table 2). The final multivariable genotype risk prediction model consisted of the anterobasal hook, multiple myocardial crypts, RV/LV ratio, MWT/PWT ratio, and MWT/LVM_i,n_. In the table, the optimal cut-off according to Youden's index is shown for each numerical variable in the model. The final model achieved an area under the ROC curve of 0.92 [95% confidence interval 0.87–0.97]. In order to determine the influence of different MRI scanners used in this study, a sensitivity analysis was performed only including G+/LVH- subjects (*n* = 44) and healthy controls (*n* = 40) that were scanned on the same MRI system. A consistent result was found with an area under the ROC curve of 0.91 [95% confidence interval 0.85–0.96]. Furthermore, a score system was constructed based on the optimal cut-offs for each variable in the model. A score of ≥3 was present only in G+/LVH- subjects, identifying 56% of the G+/LVH- population. A score of 0 excluded the possibility of a pathogenic variant (Figure 2). Assigning different weights (including equipotent distribution) did not provide additional predictive ability. There were a total of 10 subjects with family members in the cohort (5 pairs of 2 family members). To account for the potential effect of family relatedness, we repeated the analyses by excluding all but one random subject per family (*n* = 52), which yielded consistent results (data not shown).

**Table 2:** Results of logistic regression analysis for genotype-positive status.

	**Univariable analysis**		**Multivariable analysis**		**Cut-off**	**Score**
	**Odds ratio [95% CI]**	***p***	**Odds ratio [95% CI]**	***p***		
**Demographics**						
Age (years)	0.99 [0.96–1.02]	0.76				
Male sex	1.30 [0.56–3.01]	0.54				
Body mass index (kg/m^2^)	1.11 [1.00–1.23]	0.05				
Caucasian ethnicity	0.81 [0.58–1.12]	0.20				
**CMR characteristics**						
Left atrial volume (ml/m^2^)	0.99 [0.96–1.03]	0.59				
LV end-diastolic volume (ml/m^2^)	0.99 [0.95–1.02]	0.43				
LV end-systolic volume (ml/m^2^)	0.95 [0.89–1.01]	0.09				
LV ejection fraction (%)	1.08 [1.00–1.18]	0.06				
LV mass (g/m^2^)	0.90 [0.85–0.95]	<0.0001				
RV end-diastolic volume (ml/m^2^)	0.96 [0.93–0.99]	<0.01				
RV end-systolic volume (ml/m^2^)	0.92 [0.88–0.96]	<0.001				
RV ejection fraction (%)	1.20 [1.10–1.33]	<0.0001				
RV/LV ratio (0.1 increase)	0.40 [0.23–0.64]	<0.0001	0.36 [0.17–0.69]	<0.01	<1.1	1
Native T1 septum (ms)	1.01 [0.99–1.02]	0.06				
ECV basal septum (%)	1.01 [0.88–1.19]	0.85				
Crypts	5.82 [2.19–17.96]	<0.001				
≥2 crypts	15.86 [1.91–2068.46]	<0.01	10.35 [1.02–1410.39]	<0.05	n/a	2
Ratio of non-compacted*/*compacted myocardium >2.3	1.69 [0.48–5.92]	0.41				
Hook-shaped thickening basal anterior wall	27.00 [3.41–3491.40]	<0.001	19.83 [1.68–3095.67]	<0.05	n/a	2
AMVL length (mm/m^2^)	0.94 [0.75–1.17]	0.57				
Interventricular septum (mm)	0.99 [0.76–1.30]	0.96				
Maximal wall thickness (mm)	1.29 [0.97–1.73]	0.08				
Posterior wall thickness (mm)	0.54 [0.36–0.75]	<0.001				
Interventricular septum/posterior wall ratio	21.86 [3.56–183.89]	<0.001				
MWT/PWT ratio (0.1 increase)	1.47 [1.23–1.82]	<0.0001	1.40 [1.12–1.87]	<0.01	>1.4	1
MWT/LVM_i,n_ (1 increase)	1.89 [1.42–2.67]	<0.0001	1.68 [1.15–2.65]	<0.01	>11.1	1

*Logistic regression results identifying predictors of G+ status, with optimal cut-offs (Youden's index) and corresponding score. Anterobasal hook and multiple myocardial crypts were given double weight in line with high coefficients compared to other dichotomized variables (not shown)*.

*G+, Genotype-positive; LV, Left ventricle; LVM_i,n_, Left ventricular mass indexed by body surface area and normalized by sex; MWT, Maximal wall thickness; PWT, Posterior wall thickness; RV, Right ventricle*.

## Discussions

In this study, we assessed morphological, volumetric, and functional differences between a cohort of G+/LVH- subjects and healthy controls. In addition, we examined whether CMR-derived variables are useful for the prediction of pathogenic DNA variants. The main findings are that the presence of multiple crypts and anterobasal hook only occurred in G+ subjects, and that a simple score system incorporating these and other CMR-derived myocardial morphological features could identify G+/LVH- subjects correctly. This study therefore provides additional evidence with respect to the prediction of G+ status in subjects without LVH using CMR.

The diagnosis of HCM is based on a MWT ≥ 13 mm in first-degree relatives of patients with unequivocal disease (1). CMR has proven to be an excellent modality for accurate wall thickness measurement. Since our study population consists of G+/LVH- subjects, MWT as parameter for the prediction of the presence of a pathogenic DNA variant cannot be included in our score system. Therefore, we studied other morphological and functional features for the prediction of the presence of pathogenic DNA variants.

It is known that CMR is able to detect important pre-clinical expressions of HCM such as myocardial crypts. Although these myocardial crypts are known to be a non-specific myocardial feature for HCM, multiple studies have investigated its prevalence in this population. Varying prevalence rates were found, that can be explained by the use of different definitions of a myocardial crypt, and the use of non-standard imaging planes (i.e., modified 2 chamber view) to detect small structural abnormalities (5, 7, 17). Nevertheless, these studies show that the presence of multiple myocardial crypts is highly suspect for pathogenic DNA gene variants. Our study confirms this finding, since multiple myocardial crypts only occurred in our G+/LVH- subjects (5, 7, 18). In addition, several other studies showed an elongated AMVL can be regarded as a predictor for G+ status, although other studies, including our own, contradict this relationship (9, 19).

Another important morphological feature that we observed in our cohort was a prominent anterobasal hook-shaped configuration, which has been described in patients diagnosed with HCM, but as far as we know not in G+/LVH- subjects (15). In HCM patients, the initial hypertrophic response in the LV is typically expected to occur in the basal septum due to its increased wall stress, owing to a larger radius and the influence of non-basal LV contraction and RV pressure (20). We hypothesize that this hook represents the area of the initial hypertrophic response, which assumes that G+/LVH- subjects already experience elevated loading conditions or respond pathologically to normal loading conditions. The altered (hypertrabeculated) tissue outside of the septum offers a potential explanation, as this could redistribute wall stress toward the septum similar to observations in apical trabeculation in left-ventricular non-compaction (21). The resulting focal hypertrophy and myocardial thinning may also explain the significantly lower LVM_i_ in G+/LVH- subjects in our study. None of the aforementioned studies comparing G+/LVH- subjects and healthy controls found a significant difference in LVM, although measurement differences complicate comparisons (5, 7).

In the past decade, pre- and post-contrast T1 mapping has emerged as an important tool for tissue characterization (22). However, only one study has investigated this technique in G+/LVH- subjects, which demonstrated a significant increase in ECV in G+/LVH- subjects in comparison to healthy controls (23). In our study, no differences in native T1 values and/or ECV were found, although there was a trend to higher native T1 values in G+/LVH- subjects. This could be due to the relatively small sample size of healthy controls and the range of ECV values in their study (Ho et al.: G+/LVH- subjects: *n* = 29, ECV 33 ± 1, range 23–38 vs. healthy controls: *n* = 11, ECV 27 ± 1, range 24–31; present study: G+/LVH- subjects: *n* = 21, ECV 29 ± 4, range 22–37 vs. healthy controls: *n* = 26, ECV 29 ± 4, range 21–38) (23). However, it would be questionable if a small but significant difference in T1 or ECV values would be of clinical importance.

Multivariable linear regression analysis was performed using both existing and novel CMR predictors for G+ status. This showed that the anterobasal hook, multiple crypts, and parameters based on wall thickness were independent predictors for pathogenic DNA variants carriers, with the presence of an anterobasal hook being the strongest predictor of G+ status. Some differences with the study of Captur et al. were found: AMVL length and LV ESV were not identified as predictors in our study. Our result with respect to AMVL length was confirmed by a previous study in our center, which also showed no difference in AMVL length between 133 G+/LVH- subjects and 135 healthy controls on echocardiography (9). Moreover, differences in absolute LV ESV were comparable between our study and that of Captur et al., showing a similar trend with lower LV ESV in G+/LVH- subjects (5). However, this variable was not found to be a significant predictor for G+ status in both univariable and multivariable linear regression. Conversely, the presence of multiple crypts was the only variable that was a predictor in both studies, although most variables in our model have not been investigated in other studies (5). This stresses the need for further studies to confirm our findings in larger samples. Attractive clinical features to investigate in future studies include electrocardiographic variables together with existing and novel echocardiographic and CMR indices. Furthermore, recent research with artificial intelligence demonstrated a superior performance in the prediction of G+ status in patients with HCM compared to conventional scoring systems (24, 25). Future research is needed to show whether this application could be useful in G+ subjects without LVH as well.

## Limitations

It should be noted that our study is subject to limitations. Firstly, our study is hampered by a relatively small sample size and lacks external validation. Future studies are needed to externally validate our model. Secondly, the control group was not formally genotyped, and the presence of pathogenic DNA variants in this control group can therefore not be completely excluded. However, given the low number of sarcomere gene DNA variants in the general population, we believe the potential influence on our results will be very limited at best. Thirdly, CMR was not performed on one single MRI system, although 91% of the total number of examinations were performed on the same scanner. This may have influenced the results. Although in a sensitivity analysis only including these 91% of the scans, the performance of our model was consistent. Fourthly, no difference in hypertrabeculation according to the Petersen criteria was found between G+/LVH- subjects and healthy controls but fractal analysis for the quantitative measurement of trabeculation was not performed. Finally, the presence of LGE was only examined in G+/LVH- subjects, as this sequence was not performed in healthy controls. Therefore, this variable could not be included in our score system to identify G+ status.

## Conclusions

A simple score system incorporating CMR-derived myocardial morphological features correctly identified 56% of G+ subjects. Our results provide further insights into the wide phenotypic spectrum of G+/LVH- subjects and demonstrate the utility of several novel morphological features. Specifically, quantification of relative hypertrophy (or rather, thinning) is valuable even in the absence of HCM, and the presence of an anterobasal hook strongly suggests the presence of sarcomere gene variants. If genetic testing for some reason cannot be performed, CMR and our purposed score system can be used to detect possible G+ carriers and to aid planning of the control intervals. However, it should be considered that HCM most commonly develop in mid-life and imaging features may change over time, therefore a CMR without abnormalities especially in young relatives cannot be used to exclude the possibility of developing HCM later in life.

## Data Availability Statement

The original contributions presented in the study are included in the article/Supplementary Material, further inquiries can be directed to the corresponding author/s.

## Ethics Statement

The studies involving human participants were reviewed and approved by the local Institutional Review Board of the Erasmus Medical Center (MEC-2014-096). The patients/participants provided their written informed consent to participate in this study.

## Author Contributions

NV and RH: analysis and interpretation of data, drafting of the manuscript, and final approval of the manuscript. HH, RB, MS, and JV: revising manuscript critically and final approval of the manuscript. AS, MM, and AH: conception and design, revising manuscript critically, and final approval of the manuscript. All authors contributed to the article and approved the submitted version.

## Conflict of Interest

The authors declare that the research was conducted in the absence of any commercial or financial relationships that could be construed as a potential conflict of interest.

## Publisher's Note

All claims expressed in this article are solely those of the authors and do not necessarily represent those of their affiliated organizations, or those of the publisher, the editors and the reviewers. Any product that may be evaluated in this article, or claim that may be made by its manufacturer, is not guaranteed or endorsed by the publisher.

## Abbreviations

AMVL: Anterior mitral valve leaflet length
CMR: Cardiovascular Magnetic Resonance
ECV: Extracellular volume
G+: Genotype positive
HCM: Hypertrophic cardiomyopathy
IVS: Interventricular septum
LV: Left ventricle
LVH: Left ventricular hypertrophy
LVM: Left ventricular mass
LVM_i,n_: Left ventricular mass indexed by body surface area and normalized by sex
MWT: Maximal wall thickness
PWT: Posterior wall thickness
ROC: Receiver operator characteristic
RV: Right ventricle
SA: Short-axis.
