# Supplementary material for: Novel Morphological Features on CMR for the Prediction of Pathogenic Sarcomere Gene Variants in Subjects Without Hypertrophic Cardiomyopathy
Source: Front Cardiovasc Med. 2021 Sep 17;8:727405. doi: 10.3389/fcvm.2021.727405 (PMC8484536; doi:10.3389/fcvm.2021.727405)
Supplement: Supplementary file 1 [file Table_1.docx]

| G+/LVH- subjects | Genotype | Base | Amino-acid |
| --- | --- | --- | --- |
| 1 | MYBPC3 | c.1624+1G>A | p.(?) |
| 2 – 14 | MYBPC3 | c.2373dup | p.(Trp792fs) |
| 15 – 21 | MYBPC3 | c.2864_2865del | p.(Pro955fs) |
| 22 – 23 | MYBPC3 | c.442G>A | p.(Gly148Arg) |
| 24 | MYBPC3 | c.3065G>C | p.(Arg1022Pro) |
| 25 – 27 | MYBPC3 | c.654+1G>A | p.(?) |
| 28 | MYBPC3 | c.688del | p.(Gln230fs) |
| 29 – 35 | MYBPC3 | c.2827C>T | p.(Arg943*) |
| 36 | MYBPC3 | c.2308G>A | p.(Asp770Asn) |
| 37 | MYBPC3 | c.2413+1del | p.(?) |
| 38 | MYBPC3 | c.2149-2del | p.(?) |
| 39 | MYBPC3 | c.1831G>A | p.(Glu611Lys) |
| 40 | MYBPC3 | c.927-2A>G | p.(?) |
| 41 | MYBPC3 | c.932C>A | p.(Ser311*) |
| 42 | MYBPC3 | c.3776del | p.(Gln1259fs) |
| 43 – 45 | MYH7 | c.1727A>G | p.(His576Arg) |
| 46 | MYH7 | c.976G>C | p.(Ala326Pro) |
| 47 | MYH7 | c.4377G>T | p.(Lys1459Asn) |
| 48 | MYH7 | c.1816G>A | p.(Val606Met) |
| 49 | MYH7  MIB1 | c.5135G>A  c.2530_2532delinsC | p.(Arg1712Gln) p.(Ser844fs) |
| 50 – 52 | MYL2 | c.64G>A | p.(Glu22Lys) |
| 53 | MYL2 | c.403-1G>C | p.(?) |
| 54 | MYL2 | c.376C>T | p.(Gln126*) |
| 55 | MYL2  CALR3 | c.376C>T  c.564del | p.(Gln126*)  p.(Gln189fs) |
| 56 | ACTN2 | c.2569G>C | p.(Asp857His) |
| 57 | TNNT2 | c.421del | p.(Arg141Glyfs) |
| G+, Genotype-positive; LVH- , Left ventricular hypertrophy negative. | | | |
